# Alterations in intracellular potassium concentration by HIV-1 and SIV Nef

**DOI:** 10.1186/1743-422X-5-60

**Published:** 2008-05-19

**Authors:** Bongkun Choi, Cesar D Fermin, Alla M Comardelle, Allyson M Haislip, Thomas G Voss, Robert F Garry

**Affiliations:** 1 Department of Microbiology and Immunology, Tulane University Health Sciences Center, New Orleans, LA 70112, USA; 2 College of Veterinary Medicine, Nursing & Allied Health (CVMNAH), Tuskegee University, Tuskegee, AL 36088, USA; 3 Departments of Environmental Medicine, Pathology, and Medicine, New York University School of Medicine, Tuxedo, NY 10987, USA

## Abstract

**Background:**

HIV-1 mediated perturbation of the plasma membrane can produce an alteration in the transmembrane gradients of cations and other small molecules leading to cell death. Several HIV-1 proteins have been shown to perturb membrane permeability and ion transport. *Xenopus laevis *oocytes have few functional endogenous ion channels, and have proven useful as a system to examine direct effects of exogenously added proteins on ion transport.

**Results:**

HIV-1 Nef induces alterations in the intracellular potassium concentration in CD4+ T-lymphoblastoid cells, but not intracellular pH. Two electrode voltage-clamp recording was used to determine that Nef did not form ion channel-like pores in *Xenopus *oocytes.

**Conclusion:**

These results suggest that HIV-1 Nef regulates intracellular ion concentrations indirectly, and may interact with membrane proteins such as ion channels to modify their electrical properties.

## Introduction

During primary infection by HIV-1 or simian immunodeficiency virus (SIV) there is a rapid and nearly complete depletion of the mucosal CD4+ T cell population [1]. This initial phase is followed by a prolonged phase in which there is a gradual decline in the overall numbers of peripheral CD4+ T cells, which appears to reflect accelerated rates of cell death and replacement [2]. Direct HIV-1 mediated cell killing appears to be a major factor in both phases of CD4+ T-lymphocyte loss in AIDS or simian AIDS (SAIDS), although immune dysregulation and other factors also contribute [3,4]. Homostatic mechanisms attempting to replenish the CD4+ T cell pool eventually fail, leading to collapse of the naïve T cell regenerative potential and ultimately to immune system collapse [5]. Direct cytopathic effects mediated by HIV-1 or virion components may also be involved in other aspects of lentivirus pathogenesis, such as the induction of neurological dysfunctions [4,6,7]. HIV-1 mediated perturbation of the cellular membranes can produce an alteration in the transmembrane gradients of cations and other small molecules leading to cell death by lysis, cell-cell fusion, apoptosis and necrosis [8-10]. Acute cytopathic infection by HIV-1 increases the intracellular concentrations of sodium and potassium, but decreases intracellular pH [3,11,12]. Several HIV-1 proteins alter cellular electrophysiological properties. Vpr causes a large inward current and cell death in cultured hippocampal neurons [13]. Vpu forms cationic channels [14] and induces a K+ conductance in Xenopus oocytes [15], while Tat blocks L-type Ca2+ channels in dendritic cells [16]. The surface glycoprotein (SU) of HIV-1 activates the Na+/H+ antiport and K+ conductance in astrocytes [17]. The lentivirus lytic peptide domains of the HIV and SIV transmembrane glycoprotein (TM) also alter the permeability of cell membranes [18,19].

HIV-1 Nef is encoded by an open reading frame located at the 3' end of the viral genome, partially overlapping the 3' long terminal repeat (LTR) and conserved in all strains of HIV-1, HIV-2, and SIV. This protein is synthesized in every stage of the viral replication cycle, and is associated with cellular membranes via an N-terminal myristic acid [20,21]. Nef is an important regulator in the development of AIDS pathology. It down-regulates both the surface expression of CD4, the primary receptor for HIV-1 in T lymphocytes and macrophages [22], and MHC class I molecules [23]. Nef also enhances viral infectivity during the process of virion assembly and upregulates viral replication both in cell culture and in animal systems [20,24]. Nef inhibits a large-conductance potassium channel in human glial cells [7]. This study was undertaken to further investigate the possible role of Nef protein in HIV-mediated membrane modification using recombinant HIV-1 and SIV Nef proteins and *Xenopus *oocytes, a well-characterized system to evaluate effects of exogenously added proteins on ion transport.

## Results

### Alteration of intracellular potassium concentrations by recombinant Nef

**Figure 1:**
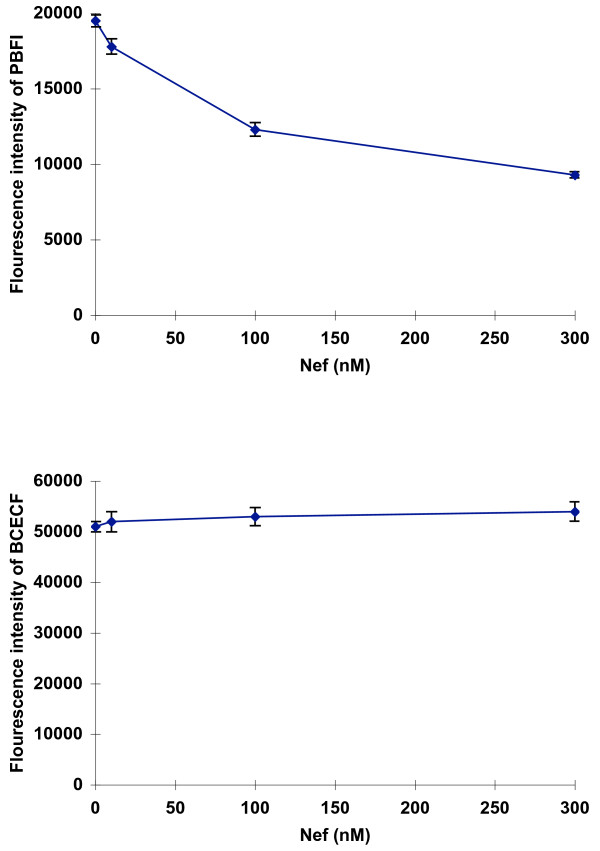
**Alterations in intracellular K+ in T-lymphoblastoid cells incubated with recombinant HIV-1 and SIV Nef protein.** H9 cells were loaded either with the K+ sensitive fluorescent indicated PBFI-AM (panel A) or the pH sensitive fluorescent indicator BCECF-AM (Panel B), then incubated with various concentrations of recombinant HIV-1 or SIV Nef protein for 15 min at 37°C, and fluorescence intensity was measured by using a fluorescence concentration analyzer. Each data point represents the mean ± standard error of eight independent determinations.

To quantify the effect of Nef on intracellular potassium concentrations ([K+]), RH9 cells from a CD4+ T-lymphoblastoid cell line were loaded with an ion-sensitive fluorescent dye, potassium-binding benzofuran isophthalate-acetoxymethyl ester (PBFI-AM) and then incubated with various concentrations of recombinant HIV-1 Nef for 15 min at 37°C. K+ fluorescence intensity was monitored using a fluorescence concentration analyzer. Incubation with HIV-1 Nef resulted in a decrease of intracellular potassium concentration in a dose dependent manner (Fig. 1A). RH9 cells incubated with recombinant SIV Nef also reduced intracellular [K+] (not shown). In contrast, H9 cells incubated with Nef maintained intracellular pH at a similar level to that of mock-infected H9 cells throughout various concentrations of Nef in the medium (Fig 1B).

**Figure 2:**
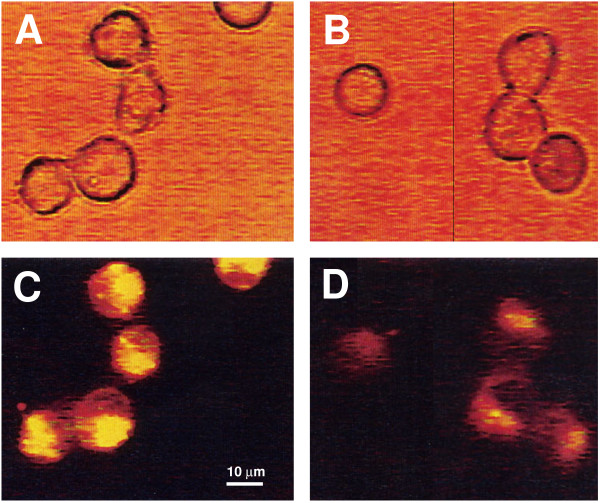
**Fluorescent and light microscopic analysis of alteration in intracellular K+ concentrations in H9 cells treated with recombinant Nef.** H9 cells were loaded with the fluorescent indicator PBFI-AM and incubated with 300 nM recombinant Nef protein or with control medium without Nef for 15 min at 37°C. Control cells (A and C) and cells incubated with Nef protein (B and D) were examined by light (A and B) and fluorescent (C and D) microscopy.

The change in intracellular K+ concentration is not due to a nonspecific quenching of fluorescence as a consequence of incubation with Nef. Examination of representative light (Fig. 2A and 2B) and fluorescence microscopic (Fig 2C and 2D) images confirmed the results using fluorescence concentration analysis of cell populations. Relative to mock-infected RH9 cells (Fig. 2C), RH9 cells incubated with Nef showed a decrease in PBFI fluorescence emission (yellow: a higher [K+], red: lower ([K+]), indicating a decrease in intracellular K+ concentration.

### Recombinant Nef does not alter transmembrane currents of *Xenopus laevis *oocytes

**Figure 3:**
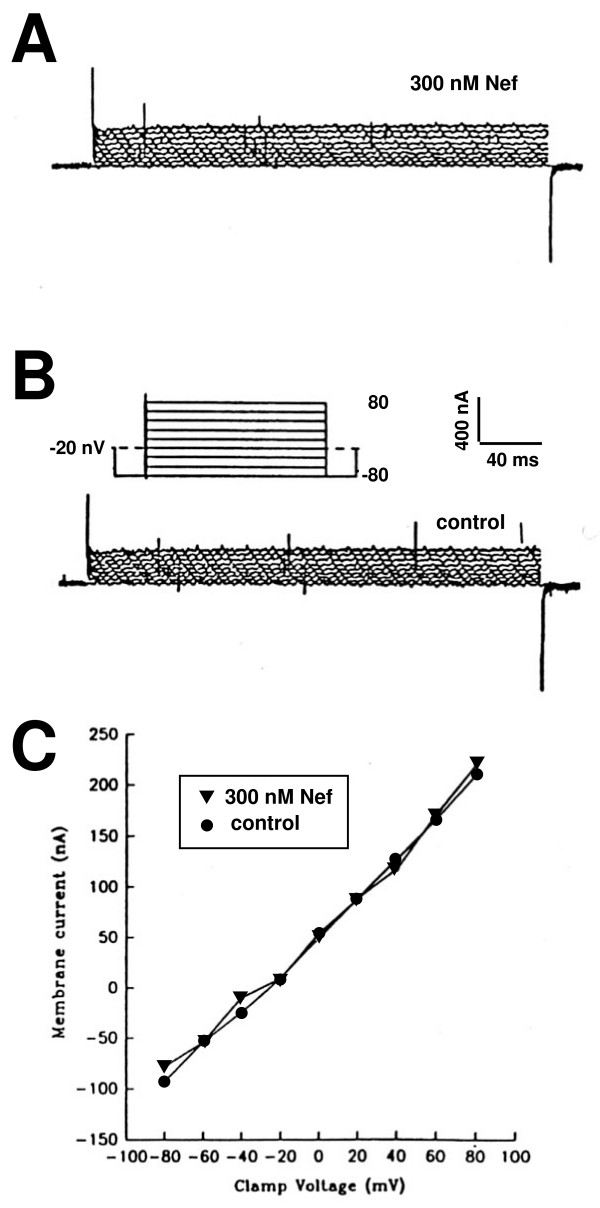
**Membrane currents in *Xenopus *oocytes exposed to Nef protein.** (A) 300 nM recombinant Nef, (B) control oocyte Panel C: current:voltage plots determined from data recorded in panel A and B.

To investigate the interaction of Nef with the plasma membrane, recombinant Nef was incubated with *Xenopus laevis *oocytes, and two electrode voltage-clamp recording was performed. A useful property of *Xenopus *oocytes is that these cells have few endogenous membrane ion channels to interfere with measurement of potential pore-forming proteins [25]. HIV-1 Lentivirus lytic peptide (LLP-1) or the bee venom peptide melittin that are known to form ion channel-like pores in membranes induced increase in transmembrane currents of Xenopus oocytes. Nef failed to induce a current differing from that of untreated oocytes within 30 sec even at 300 nM, a concentration shown to be cytostatic (Fig. 3). No increase in transmembrane currents was observed in measurements at 5 or 30 min after addition of 300 nM of Nef, unlike LLP-1 or bee venom peptide melittin [25]. These results suggest that Nef does not form an ion-channel-like structure, but rather may interact with ion channels to affect K+ levels in other cells, such as CD4+ lymphocytes.

## Discussion

The results presented here add to previous studies suggesting that alteration of membrane ion transport and permeability are important mechanisms for HIV-1 cytopathogenesis. The intracellular K+ concentration is reduced in T-lymphoblastoid cells in the presence of exogenously added Nef. This alteration is selective, since intracellular pH was unaffected. Potassium channels are among the primary machinery mediating ion homeostasis in human lymphocytes. Therefore, modulation of intracellular K+ concentration by Nef may be involved in HIV-1 mediated cytopathogenesis. Previously, soluble Nef was shown to be cytostatic and cytotoxic against both CD4+ T cells and monocytoid cell lines [25].

Several viral proteins, including influenza A virus M2, influenza B virus NB, HIV-1 Env, Vpu, and Vpr, induce alterations in cellular membrane permeability or ion transport [26]. The classic example of a virally-encoded ion channel (viroporin) is the M2 protein of influenza virus, which forms a well-characterized proton channel and is also a target for an important class of anti-influenza drugs [27]. HIV-1 Vpu structurally resembles M2 and forms a pH-independent ion channel in lipid bilayers [28]. Vpr also appears to have ion channel like properties [13]. The LLP domains of HIV TM also have the capacity to interact with membranes and alter permeability [18,29]. In contrast to these other HIV-1 proteins, Nef does not appear to directly modify permeability or form an ion-channel-like structure. Rather, Nef may interact with ion channels and modify their electrical properties. Previous studies by Kort and others are consistent with this hypothesis. Nef has been demonstrated to inhibit a large-conductance potassium channel in human glial cells and to inhibit the activity of a Ca2+ -dependent K+ channel in a T lymphocyte cell line [7]. The structural similarity of Nef to snake neurotoxins is additional evidence for the role of Nef in ion channel modulation, and is suggestive of a role of Nef in AIDS-associated neuropathologies [30]. Snake neurotoxins are known to interact with ion channels. Direct effects of Nef on cell membranes are not excluded by our studies. Indeed, Nef has also been shown to cause perturbation and fusion of artificial membranes and lysis of human lymphocytes and red blood cells [31]. It has been observed that exogenous Nef causes cell death in yeast and bacterial cell due to permeabilization of the cell membranes [32].

This effect of Nef proteins shown in this study requires the presence of the protein extracellularly. This could be achieved by lysis of HIV-infected cells in patients. Antibodies against Nef have been detected in HIV-infected individuals [33], suggesting the extracellular presence of Nef *in vivo*. The concentrations of Nef used in the current experiments are likely to be physiologically relevant. A high percentage of sera samples from HIV-1 positive individuals contained Nef at a concentration 5–10 ng/ml (approximately 0.2–0.4 nM) that was shown to be cytostatic [33]. This implies that effective Nef concentrations achieved locally in tissues where HIV-1 replicates, such as intestine, lymph node, and brain, prior to dilution in the vasculature might be several orders of magnitude higher consistent with concentrations (mid nM) shown here to affect [K+] in lymphoid cells.

The overall affect of HIV-1 infection on K+ transport is likely to be complex. Our previous studies indicated that during cytopathic infection intracellular [K+] increases [12,34]. Furthermore, increased intracellular [K+] in infected cells enhances HIV-1 replication and accelerates cell killing, particularly the CPE described as "ballooning degeneration" [34]. Therefore, it is possible that Nef may function in a regulatory or compensatory role for ion fluxes mediated by other HIV-1 proteins.

## Materials and methods

### Cells, virus and recombinant proteins

Cells of the RH9 subclone of the CD4+ human T-lymphoblastoid cell line RH9 were the kind gift of Dr. Suraiya Rasheed (University of Southern California), and were maintained in RPMI 1640 supplemented with 10% fetal bovine serum[6] (GIBCO, Long Island, NY), penicillin (100 U/ml) and streptomycin (100 μg/ml). Oocytes were removed from anesthetized *Xenopus laevis*, and prepared for electrophysiological evaluation as previously described [18]. All animal experiments were evaluated and approved by the Tulane University Health Sciences Center Institutional Animal Care and Use Committee. Recombinant HIV-1 and SIV Nef was obtained from the NIH AIDS Research & Reference Reagent Program.

### Measurement of intracellular potassium and pH concentrations with fluorescent indicators

Potassium-binding benzofuran isophthalate-acetoxymethyl ester (PBFI-AM) was used for fluorometric determination of ion ceoncentrations in intact, living cells [12]. Intracellular pH in RH9 cells were measured using the pH-sensitive fluorescence dye 2'7'-bis(2-carboxyethyl)-5(6)- carboxyfluorescein-acetoxymethyl ester (BCECF-AM) [11]. The acetoxymethyl ester group linked to each dye renders the molecule uncharged and thereby able to permeate living cell membranes. Once inside the cell, the lipophilic blocking groups are cleaved by endogenous cellular esterases, resulting in a charge free acid that is unable to pass through the cell membrane. Upon binding by target ion, the excitation maximum of this fluorescent indicator shifts to shorter wavelengths, causing a large alteration in the ratio of energy absorbed. This induces a 2.5 fold enhancement of fluorescent intensity with little change in the emission maximum. After adding PBFI- AM or BCECF-AM at final concentration of 10 μM or μM, cells were incubated for 2 hours or 30 min, respectively. Cells labeled with PBFI or BCECF were plated into 96-well Fluoricon GF assay plate (10,000 cells per well) fitted with a glass fiber filter (1.0 μm pore size), and washed three times. The FCA allows vacuuming of solutions through the well thereby concentrating cells at the bottom. To prevent cells from clogging the filter membrane, inert polystyrene particles (3.3 μm diameter) were added to each well prior to addition of cells. PBFI and BCECF fluorescence intensity were determined with the photomultiplier gain set at 100. 8 replicates were quantified for each sample. Microscopic analysis of cells loaded with fluorescent indicator of K+ was performed as previously described [11,12].

### Two-electrode voltage clamping

The whole-cell currents were measured with a GeneClamp 500 amplifier using two microelectrodes as previously described [25]. Currents in oocytes exposed to 300 nM recombinant Nef were determined by holding the membrane voltage to a set point of -20 mV, then changing the membrane voltage stepwise from -80 to +80 mV in 20 mV increments by 280- msec pulses with 60-msec prepulse at -80 mV and a 60 msec afterplus at -80 mV. Measurements were conducted within 30 sec of adding Nef to the circulating bath.

## Conclusion

HIV-1 Nef induce alterations in the intracellular [K+] in CD4+ T-lymphoblastoid cells, but not intracellular pH. Nef proteins do not form ion channel-like pores in *Xenopus *oocytes and may regulate intracellular [K+] in CD4+ lymphocytes, and perhaps other cell types, by interacting with monovalent ion channels.

## Competing interests

The authors declare that they have no competing interests.

## Authors' contributions

BC performed all experiments with substantial help from AMC. RFG, TGV and CDF provided guidance, expertise, equipment, and funding for these experiments. All authors have read and approved this manuscript.

## References

[B1] Veazey RS, DeMaria M, Chalifoux LV, Shvetz DE, Pauley DR, Knight HL, Rosenzweig M, Johnson RP, Desrosiers RC, Lackner AA (1998). Gastrointestinal tract as a major site of CD4+ T cell depletion and viral replication in SIV infection. Science.

[B2] Cooper DA, Gold J, Maclean P, Donovan B, Finlayson R, Barnes TG, Michelmore HM, Brooke P, Penny R (1985). Acute AIDS retrovirus infection. Definition of a clinical illness associated with seroconversion. Lancet.

[B3] Garry RF, Gottlieb AA, Zuckerman KP, Pace JR, Frank TW, Bostick DA (1988). Cell surface effects of human immunodeficiency virus. Biosci Rep.

[B4] Costin JM (2007). Cytopathic mechanisms of HIV-1. Virol J.

[B5] Nishimura Y, Igarashi T, Buckler-White A, Buckler C, Imamichi H, Goeken RM, Lee WR, Lafont BA, Byrum R, Lane HC (2007). Loss of naive cells accompanies memory CD4+ T-cell depletion during long-term progression to AIDS in Simian immunodeficiency virus-infected macaques. J Virol.

[B6] Garry RF (1989). Potential mechanisms for the cytopathic properties of HIV. Aids.

[B7] Kort JJ, Jalonen TO (1998). The nef protein of the human immunodeficiency virus type 1 (HIV-1) inhibits a large-conductance potassium channel in human glial cells. Neurosci Lett.

[B8] Rasheed S, Gottlieb AA, Garry RF (1986). Cell killing by ultraviolet-inactivated human immunodeficiency virus. Virology.

[B9] Fermin CD, Garry RF (1992). Membrane alterations linked to early interactions of HIV with the cell surface. Virology.

[B10] Gatti PJ, Choi B, Haislip AM, Fermin CD, Garry RF (1998). Inhibition of HIV type 1 production by hygromycin B. AIDS Res Hum Retroviruses.

[B11] Makutonina A, Voss TG, Plymale DR, Fermin CD, Norris CH, Vigh S, Garry RF (1996). Human immunodeficiency virus infection of T-lymphoblastoid cells reduces intracellular pH. J Virol.

[B12] Voss TG, Fermin CD, Levy JA, Vigh S, Choi B, Garry RF (1996). Alteration of intracellular potassium and sodium concentrations correlates with induction of cytopathic effects by human immunodeficiency virus. J Virol.

[B13] Piller SC, Ewart GD, Premkumar A, Cox GB, Gage PW (1996). Vpr protein of human immunodeficiency virus type 1 forms cation-selective channels in planar lipid bilayers. Proc Natl Acad Sci U S A.

[B14] Schubert U, Ferrer-Montiel AV, Oblatt-Montal M, Henklein P, Strebel K, Montal M (1996). Identification of an ion channel activity of the Vpu transmembrane domain and its involvement in the regulation of virus release from HIV-1-infected cells. FEBS Lett.

[B15] Coady MJ, Daniel NG, Tiganos E, Allain B, Friborg J, Lapointe JY, Cohen EA (1998). Effects of Vpu expression on Xenopus oocyte membrane conductance. Virology.

[B16] Poggi A, Rubartelli A, Zocchi MR (1998). Involvement of dihydropyridine-sensitive calcium channels in human dendritic cell function. Competition by HIV-1 Tat. J Biol Chem.

[B17] Bubien JK, Benveniste EN, Benos DJ (1995). HIV-gp120 activates large-conductance apamin-sensitive potassium channels in rat astrocytes. Am J Physiol.

[B18] Comardelle AM, Norris CH, Plymale DR, Gatti PJ, Choi B, Fermin CD, Haislip AM, Tencza SB, Mietzner TA, Montelaro RC, Garry RF (1997). A synthetic peptide corresponding to the carboxy terminus of human immunodeficiency virus type 1 transmembrane glycoprotein induces alterations in the ionic permeability of Xenopus laevis oocytes. AIDS Res Hum Retroviruses.

[B19] Costin JM, Rausch JM, Garry RF, Wimley WC (2007). Viroporin potential of the lentivirus lytic peptide (LLP) domains of the HIV-1 gp41 protein. Virol J.

[B20] Kestler HW, Ringler DJ, Mori K, Panicali DL, Sehgal PK, Daniel MD, Desrosiers RC (1991). Importance of the nef gene for maintenance of high virus loads and for development of AIDS. Cell.

[B21] Fackler OT, Kienzle N, Kremmer E, Boese A, Schramm B, Klimkait T, Kucherer C, Mueller-Lantzsch N (1997). Association of human immunodeficiency virus Nef protein with actin is myristoylation dependent and influences its subcellular localization. Eur J Biochem.

[B22] Garcia JV, Miller AD (1991). Serine phosphorylation-independent downregulation of cell-surface CD4 by nef. Nature.

[B23] Schwartz O, Marechal V, Le Gall S, Lemonnier F, Heard JM (1996). Endocytosis of major histocompatibility complex class I molecules is induced by the HIV-1 Nef protein. Nat Med.

[B24] Jamieson BD, Aldrovandi GM, Planelles V, Jowett JB, Gao L, Bloch LM, Chen IS, Zack JA (1994). Requirement of human immunodeficiency virus type 1 nef for in vivo replication and pathogenicity. J Virol.

[B25] Cooke SJ, Coates K, Barton CH, Biggs TE, Barrett SJ, Cochrane A, Oliver K, McKeating JA, Harris MP, Mann DA (1997). Regulated expression vectors demonstrate cell-type-specific sensitivity to human immunodeficiency virus type 1 Nef-induced cytostasis. J Gen Virol.

[B26] Carrasco L (1995). Modification of membrane permeability by animal viruses. Adv Virus Res.

[B27] Lamb RA, Pinto LH (1997). Do Vpu and Vpr of human immunodeficiency virus type 1 and NB of influenza B virus have ion channel activities in the viral life cycles?. Virology.

[B28] Ewart GD, Sutherland T, Gage PW, Cox GB (1996). The Vpu protein of human immunodeficiency virus type 1 forms cation-selective ion channels. J Virol.

[B29] Plymale DR, Comardelle AM, Fermi CD, Martin DS, Costin JM, Norris CH, Tencza SB, Mietzner TA, Montelaro RC, Garry RF (1999). Concentration-dependent differential induction of necrosis or apoptosis by HIV-1 lytic peptide 1. Peptides.

[B30] Werner T, Ferroni S, Saermark T, Brack-Werner R, Banati RB, Mager R, Steinaa L, Kreutzberg GW, Erfle V (1991). HIV-1 Nef protein exhibits structural and functional similarity to scorpion peptides interacting with K+ channels. Aids.

[B31] Curtain CC, Lowe MG, Arunagiri CK, Mobley PW, Macreadie IG, Azad AA (1997). Cytotoxic activity of the amino-terminal region of HIV type 1 Nef protein. AIDS Res Hum Retroviruses.

[B32] Macreadie IG, Lowe MG, Curtain CC, Hewish D, Azad AA (1997). Cytotoxicity resulting from addition of HIV-1 Nef N-terminal peptides to yeast and bacterial cells. Biochem Biophys Res Commun.

[B33] Fujii Y, Otake K, Tashiro M, Adachi A (1996). Soluble Nef antigen of HIV-1 is cytotoxic for human CD4+ T cells. FEBS Lett.

[B34] Choi B, Gatti PJ, Haislip AM, Fermin CD, Garry RF (1998). Role of potassium in human immunodeficiency virus production and cytopathic effects. Virology.

